# Fabrication of Two-Layer Aluminum Foam Consisting of Dissimilar Aluminum Alloys Using Optical Heating

**DOI:** 10.3390/ma17040894

**Published:** 2024-02-15

**Authors:** Yoshihiko Hangai, Tatsuki Takagi, Yu Goto, Kenji Amagai

**Affiliations:** Graduate School of Science and Technology, Gunma University, Kiryu 376-8515, Japan

**Keywords:** cellular materials, composite, aluminum alloy, foam, optical heating, joining

## Abstract

Aluminum foam is a lightweight material and has excellent shock-absorbing properties. Various properties of aluminum foam can be obtained by changing the base aluminum alloy. Multi-layer aluminum foam can be fabricated by varying the alloy type of the base aluminum alloy, but with different foaming temperatures, within a single aluminum foam to achieve multiple properties. In this study, we attempted to fabricate a two-layer aluminum foam with the upper layer of a commercially pure aluminum A1050 foam and the lower layer of an Al-Si-Cu aluminum alloy ADC12 foam by using an optical heating device that can heat from both the upper and lower sides. Two types of heating methods were investigated. One is to directly stack the A1050 precursor coated with black toner on top of the ADC12 precursor and to foam it from the top and bottom by optical heating. The other is to place a wire mesh between the ADC12 precursor and the A1050 precursor and place the A1050 precursor on the wire mesh, thereby creating a space between the precursors, which is then foamed by optical heating from the top and bottom. It was shown that both precursors can be foamed and joined, and a two-layer A1050/ADC12 foam can be fabricated for both types of heating methods. In the method in which two precursors were stacked and foamed, even if the light intensity of the halogen lamps on the top and bottom were adjusted, heat conduction occurred between the stacked precursors, and the foaming of each precursor could not be controlled, resulting in tilting of the joining interface. In the method of foaming using a wire mesh with a gap between two precursors, it was found that by adjusting the light intensity, the two precursors can be foamed almost simultaneously and achieve similar pore structures. The joining interface can also be maintained horizontally.

## 1. Introduction

Aluminum foam is a lightweight material that floats on water and has excellent shock-absorbing, sound insulation, thermal insulation, and vibration damping properties [1,2,3]. Therefore, it is expected to be used for automotive and train parts for the shock-absorbing properties, construction materials for the sound and thermal insulation properties, and milling machines to improve the vibration-damping properties [4,5,6,7,8,9,10]. Aluminum foam has been attempted to be fabricated from various aluminum alloys, such as pure aluminum [11,12,13,14,15,16,17,18], Al-Cu aluminum alloy [19,20], Al-Si aluminum alloy [21,22,23,24,25], Al-Mg aluminum alloy [26,27,28,29], Al-Mg-Si aluminum alloy [23,30,31,32,33,34], Al-Zn-Mg Aluminum alloy [19,35,36], etc., and various properties can be obtained by changing the base aluminum alloy. Therefore, multi-layer aluminum foam can be fabricated by varying the alloy type of the base aluminum alloy within a single aluminum foam to achieve multiple properties. For example, a two-layer aluminum foam consisting of commercially pure aluminum A1050 foam and Al-Mg-Si aluminum alloy A6061 foam has been fabricated using the precursor method [37,38]. The precursor foaming process is a method in which aluminum mixed with a foaming agent is heated for foaming [39,40,41,42,43,44]. A1050 and A6061 precursors were joined by friction stir welding (FSW), and the joined precursors were foamed to obtain two-layer A1050/A6061 foam. When the obtained aluminum foam was subjected to a compression test, deformation began in the low-strength A1050 foam, followed by the A6061 foam. This method has also been used to fabricate multi-layer aluminum foam, such as two-layer A1050/Al-Si-Cu aluminum alloy ADC12 foam [45] and three-layer A1050/A6061/ADC12 foam [46]. However, it is difficult to simultaneously foam precursors consisting of aluminum alloys with different melting points in the precursor method. It is known that precursor foams gradually above the solidus temperature and rapidly above the liquidus temperature. When a precursor consisting of multiple alloys is heated in an electric furnace, foaming begins in the low melting point part of the precursor, followed by foaming in the high melting point part. Therefore, the low melting point part of the precursor is overheated, resulting in coarse pores, and the high melting point part of the precursor is not sufficiently foamed.

We have attempted to use optical heating for the foaming of the precursor. In optical heating, the time until the precursor foams can be varied by adjusting the light intensity or by painting the surface of the precursor black to increase light absorption [47]. In our previous study, an individual A1050 precursor with high foaming temperature and an ADC12 precursor with low foaming temperature were placed on the same plane on the left and right sides, and black toner was only applied to the surface of the A1050 precursor, which was then heated by light from above to simultaneously foam, which was then pressed immediately after foaming to form a two-layer A1050/ADC12 foam. It is known that foamed aluminum in a soft state immediately after foaming can be shaped by press forming while maintaining the pore structures [48]. In the case of two-layer A1050/ADC12 foam, the material flow caused by the pressing process eliminates the joining boundary, allowing the fabrication of a single aluminum foam. However, the above method can only fabricate thin sheets that are multilayered in the planar direction, and it is difficult to obtain samples that are multilayered in the thickness direction.

In this study, we attempted to fabricate a two-layer aluminum foam with the upper layer of an A1050 foam and the lower layer of an ADC12 foam by using an optical heating device that can heat from both the upper and lower sides. Since the solidus and liquidus temperatures of A1050 aluminum are 646 °C and 657 °C, respectively, and those of ADC12 aluminum alloy are 515 °C and 580 °C, respectively [49], the foaming temperatures are significantly different among the aluminum alloys. A1050 and ADC12 precursors were stacked on the top and bottom, the A1050 precursor was foamed by optical heating from above, and the ADC12 precursor was foamed by optical heating from below, with the amount of light adjusted independently for each. Then, A1050 foam and ADC12 foam were joined by lightly pressing in the vertical direction immediately after foaming. In this study, two types of heating methods were investigated. One was to directly stack the A1050 precursor coated with black toner on top of the ADC12 precursor and to foam it from the top and bottom by optical heating. The other was to place a wire mesh between the A1050 and ADC12 precursors and place the A1050 precursor on the wire mesh, thereby creating a space between the precursors, which was then foamed by optical heating from the top and bottom. The obtained samples were observed by X-ray computed tomography (X-ray CT) imaging and cutting, and the pore structures and joining interface of the obtained A1050/ADC12 foam were observed.

## 2. Precursor Fabrication Method

**Figure 1:**
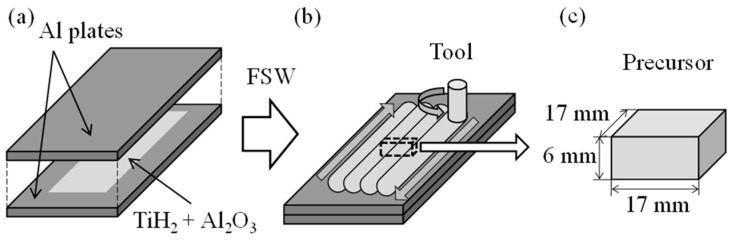
Schematic illustration of the precursor fabrication process. (**a**) A mixture of foaming agent powder and pore structures stabilizer powder was placed between the two A1050 plates. (**b**) FSW was conducted. (**c**) Precursor was obtained.

Precursors were fabricated by FSW [50]. FSW can be performed at low temperatures without melting [51,52,53,54,55,56]. Figure 1 shows a schematic illustration of the precursor fabrication process. For the fabrication of the A1050 precursor, two 210 mm × 80 mm × 3 mm thick A1050 rolled plates were prepared. A mixture of foaming agent powder and pore structures stabilizer powder was placed on a 170 mm × 20 mm area between the two A1050 plates. Titanium hydride (TiH_2_, particle size less than 45 μm) was used as the foaming agent powder and alumina (Al_2_O_3_, particle size approximately 1 μm) as the pore structures stabilizer powder. The stacked plates were placed on the FSW machine, and the tool was traversed over the area where the powder was distributed. The FSW tool was made of tool steel with a shoulder diameter of 17 mm, and the probe was M6 threaded and 4.8 mm long. The tool was traversed at a tool speed of 2200 rpm, a traversing speed of 100 mm/min, and a tilt angle of 3 degrees. Since the entire powder dispersion area cannot be stirred in a single traverse, four lines were traversed by shifting the tool perpendicularly to the traverse direction by 5 mm after each line of traversal. In order to increase the mixing of the powder, the tool was traversed over the same areas again so that the same areas were stirred twice in total. These conditions were set with reference to previous literature [50]. Precursors with dimensions 17 mm × 17 mm × 6 mm were machined from the stirred area.

The ADC12 precursor was prepared via a similar process. Only the differences from the preparation of the A1050 precursor are described below. ADC12 plates were fabricated by the die-casting method [57,58,59]. The tool was traversed at a tool speed of 1200 rpm and a tool traversing speed of 120 mm/min, and the same area was stirred four times. These conditions were set with reference to previous literature [60].

## 3. Influence of Temperature Increase Rate Caused by Differences in Optical Heating Current and Application of Black Toner

**Figure 2:**
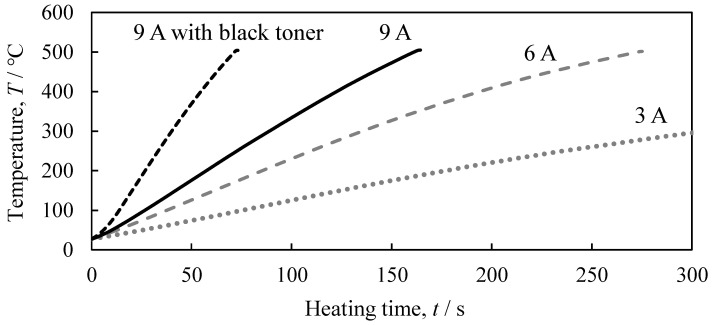
Relationship between heating time and temperature for optical heating current with 9 A, 6 A, 3 A, and 9 A with application of black toner.

We first examined variations in the rate of temperature increase caused by differences in the optical heating current and by the application of black toner. The results of heating a 17 mm × 17 mm × 6 mm dense A1050 block up to 500 °C with a thermocouple inserted in the center are shown in Figure 2. At a current of 3 A, the temperature did not reach 500 °C. The temperature increase rate increased as the current value was increased. Due to heat dissipation, the temperature rose more slowly and took more time at the lower current value. The application of a black toner can significantly increase the temperature increase rate at the same current value.

## 4. In the Case of Foaming Stacked A1050 and ADC12 Precursors

### 4.1. Heat Foaming Method

**Figure 3:**
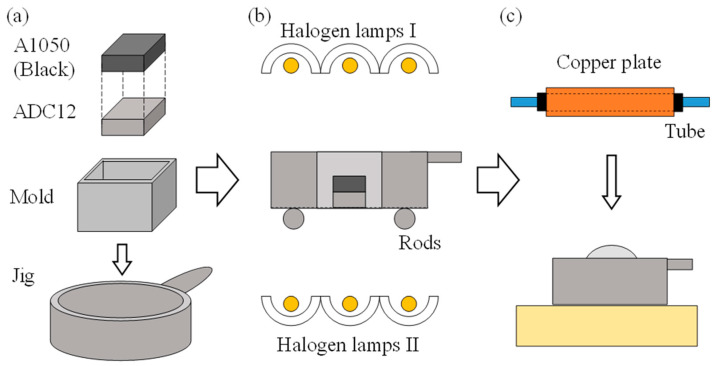
Schematic illustration of the method of heating and foaming two stacked precursors. (**a**) Preparation of precursors, mold and jig. (**b**) Optical heating. (**c**) Press forming.

**Figure 4:**
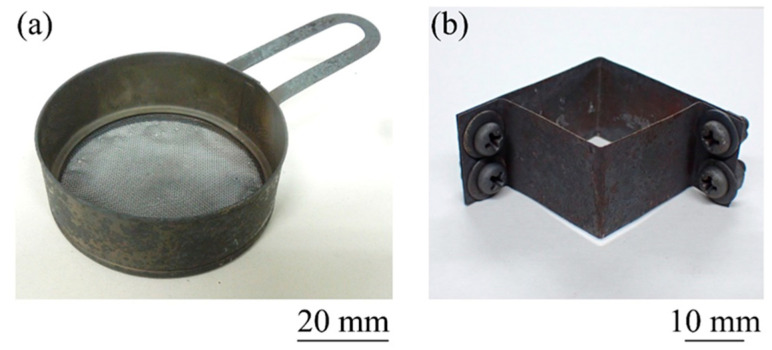
(**a**) Jig and (**b**) mold used for heating and foaming two stacked precursors.

Figure 3 shows a schematic illustration of the method of heating and foaming two stacked precursors. First, a square mold made of stainless steel sheets with a wall thickness of 0.2 mm was placed on a circular jig with a stainless steel wire mesh underside that allows light to transmit through. Figure 4 shows photographs of the jig and mold used in this study. The inside dimensions of the mold were 27 mm × 27 mm × 20 mm. In this mold, the ADC12 precursor was first placed, and then the A1050 precursor was placed on top of it. The A1050 precursor was coated with black toner to increase light absorption and foamed simultaneously with the ADC12 precursor as much as possible. Next, as shown in Figure 3b, the precursor was heated by three halogen lamps, one each from the top and bottom. “Halogen lamps I” on the upper surface were set to a constant current of 9 A and a constant voltage of 180 V per lamp to heat the A1050 precursor. “Halogen lamps II” on the underside of the ADC12 precursor were heated by varying the current by 1 A from 3 A and 60 V to 6 A and 120 V per lamp. When foaming occurred and an aluminum foam was observed from the top surface of the mold, heating was stopped, and the copper plate was pressed to the height of the mold by water cooling, as shown in Figure 3c. The upper layer of A1050 foam was pressed against the lower layer of ADC12 foam with a water-cooled copper plate to break the oxide film of each, join them metallurgically, and prevent coarsening of the pores by rapid cooling. After pressing, the aluminum foam was left to cool to room temperature and then extracted from the mold to obtain a two-layer A1050/ADC12 foam.

### 4.2. Results and Discussion

**Figure 5:**
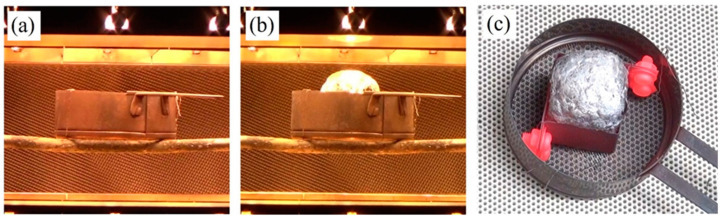
Foaming process of two stacked precursors. (**a**) Before heating. (**b**) End of heating. (**c**) Top view of foaming immediately after the heating was stopped.

Figure 5 shows the foaming process. Figure 5a is before heating, and Figure 5b is at the end of heating. Although the precursor in the mold cannot be observed during heating, heating was stopped when the aluminum foam was observed from the top of the mold, as shown in Figure 5b. Figure 5c shows a top view of the foaming immediately after the heating was stopped. The precursor is foamed in the mold, and the aluminum foam can be seen from the top of the mold. The part in red was the screw that fastened the thin stainless steel plates of the mold, which turned red during the heating process.

**Figure 6:**
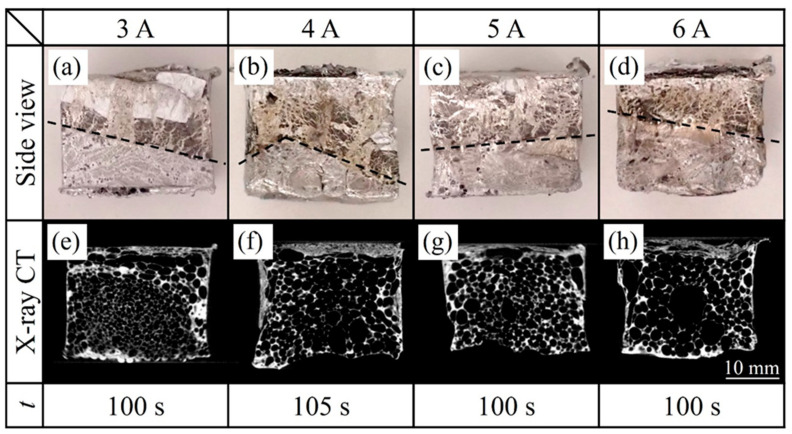
Side views of the resulting two-layer aluminum foam, an X-ray CT image of a longitudinal section near the center of the sample, and the time *t* from the start of heating the precursor to the end of the heating process for heating and foaming two stacked precursors. “Halogen lamps II” on the underside of the ADC12 precursor were varied from 3 A to 6 A.

Figure 6 shows side views of the resulting two-layer aluminum foam, an X-ray CT image of a longitudinal section near the center of the sample, and the time *t* from the start of heating the precursor to the end of the heating process. The upper and lower layers are A1050 foam and ADC12 foam, respectively, with the current of halogen lamps II on the ADC12 side varied. As shown in Figure 6a–d, it was shown that both precursors were foamed, and the A1050 foam layer and the ADC12 foam layer were joined without any gaps. No separation of the two-layer aluminum foam was observed when the obtained aluminum foam was extracted from the mold. The X-ray CT images in Figure 6e–h show that there was no clear boundary between the A1050 foam layer and the ADC12 foam layer, even in the inner part, indicating that they were joined. In the case of Figure 6e, where the current of the lower halogen lamps II was low, the ADC12 foam in the lower layer with fine pores penetrated into the A1050 foam layer in the upper layer from the lower left. The ADC12 foam is presumed to be in the initial stage of foaming due to its fine pores. The current of the halogen lamps I was high, and the A1050 precursor coated with black toner foamed first, and then the ADC12 precursor started foaming. Therefore, it is considered that the ADC12 foam that foamed later penetrated the softened A1050 foam layer, and the A1050 foam was pushed upward. The X-ray CT images in Figure 6e–h show a thin dense layer in the upper part of the A1050 layer, which is considered to be caused by excessive A1050 foaming and the beginning of a gas leakage. Furthermore, the foaming time *t* was almost the same for all samples, and the current of the upper halogen lamps I was kept constant, indicating that the heating time was dominated by the effect of the light absorption of the A1050 precursor with black toner applied. That is, the A1050 precursor foamed before the ADC12 precursor, and the heat from the A1050 precursor was transferred to the ADC12 precursor to promote foaming of the ADC12 precursor. The boundary between the A1050 foam layer and the ADC12 foam layer was inclined, as shown by the black dotted lines in Figure 6a–d. The precursors did not foam equally, and the timing of foaming differed slightly depending on the location of the precursor, which is presumed to tilt the boundary between the A1050 foam and ADC12 foam. These results indicate that the method of overlapping two precursors for foaming made it difficult to control the temperature and the start of foaming simultaneously because the two precursors were in contact and conducted heat, and it was difficult to control the boundary shape.

## 5. In the Case of Foaming with Spacing between A1050 and ADC12 Precursors Using Wire Mesh

### 5.1. Heat Foaming Method

**Figure 7:**
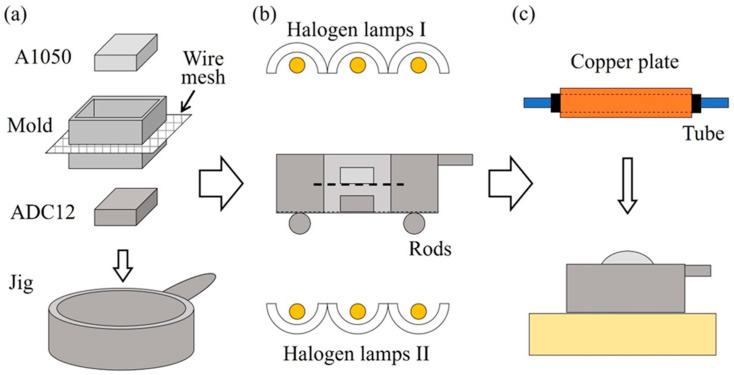
Schematic illustration of the method of heating and foaming two precursors with a gap between them. (**a**) Preparation of precursors, mold and jig. (**b**) Optical heating. (**c**) Press forming.

**Figure 8:**
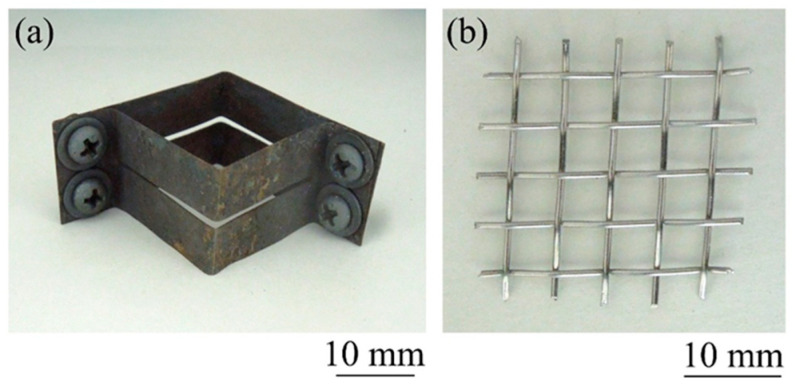
(**a**) Mold and (**b**) wire mesh used for heating and foaming two precursors with a gap between them.

Figure 7 shows a schematic illustration of the method of heating and foaming two precursors with a gap between them. First, as shown in Figure 7a, an ADC12 precursor was placed on a circular jig with a stainless steel wire mesh underside, followed by a square mold made of stainless steel sheets with a wall thickness of 0.2 mm, which was also used in Section 4. As shown in Figure 8a, this mold has a slit in the middle in the height direction, and a wire mesh can be installed, as shown in Mold in Figure 7a. The wire mesh, as shown in Figure 8b, was made of stainless steel with a wire diameter of 0.75 mm, an opening of 4.33 mm, and an opening ratio of 72.6%. Next, an A1050 precursor was placed on this wire mesh. This allows for spacing between the precursors, and unlike the case of stacking the precursors shown in Section 4, heat from each precursor was not transferred to the other precursor, and it is expected that the temperature of the precursors can be controlled independently. Next, as shown in Figure 7b, the precursors were heated by three halogen lamps, one each from the top and bottom. The upper “Halogen lamps I”, which heat the A1050 precursor, were set to a constant current of 9 A and voltage of 180 V per lamp. For the lower “Halogen lamps II”, which heat the ADC12 precursor, the current was varied in 1 A increments from 1 A and 20 V to 6 A and 120 V per lamp. When foaming occurred and aluminum foam was observed from the top surface of the mold, heating was stopped, and the copper plate was pressed to the height of the mold by water-cooling, as shown in Figure 7c. After press forming, the aluminum foam was cooled to room temperature and extracted from the mold to obtain a two-layer A1050/ADC12 foam.

Note that the wire mesh between the two precursors was embedded into the two-layer aluminum foam when the A1050 and ADC12 precursors were foamed and joined. It is known from previous studies that the tensile strength can be increased by embedding wire mesh in aluminum foam, which has a low tensile strength [61]. Therefore, it is expected that aluminum foam with superior tensile properties can be obtained even in two-layer aluminum foam.

### 5.2. Results and Discussion

**Figure 9:**
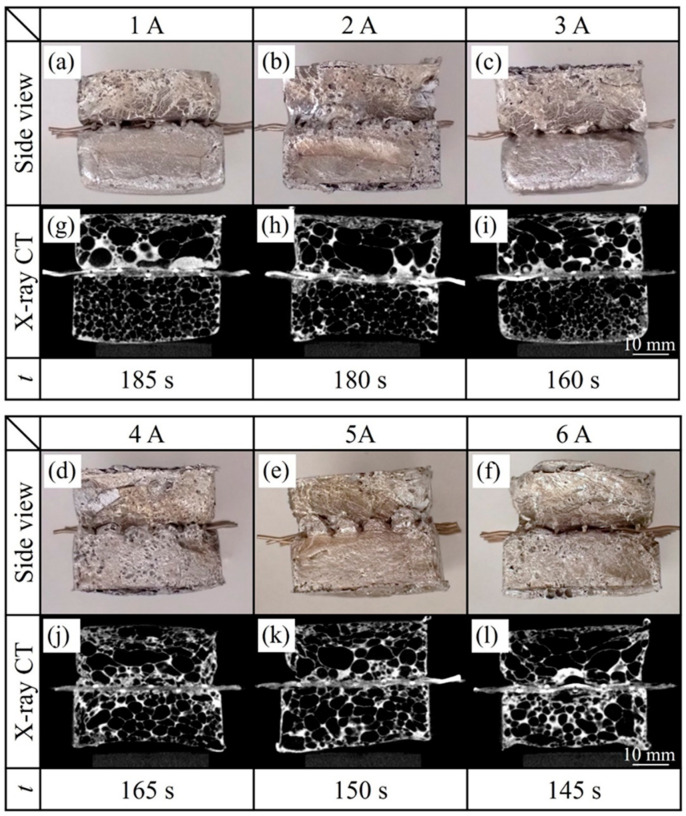
Side views of the resulting two-layer aluminum foam, an X-ray CT image of a longitudinal section near the center of the sample, and the time *t* from the start of heating the precursor to the end of the heating process for heating and foaming two precursors with a gap between them. “Halogen lamps II” on the underside of the ADC12 precursor were varied from 1 A to 6 A.

Figure 9 shows side views of the resulting two-layer aluminum foam, X-ray CT images of a longitudinal section near the center of the samples, and the time *t* from the start of heating the precursor to the end of the heating process. The upper and lower layers are A1050 foam and ADC12 foam, respectively, where the current of “Halogen lamps II” on the ADC12 side varied. As shown in Figure 9a–f, although there were some gaps between the upper layer of the A1050 foam and the lower layer of the ADC12 foam, they were joined. No separation was observed during extraction from the mold or by cutting, as described below. The X-ray CT images in Figure 9g–l show that the A1050 and ADC12 foam layers were kept nearly parallel by the wire mesh and joined near the center of the two-layer aluminum foam obtained. Unlike the case where the precursors were placed on top of each other as described in Section 4, the joining interface was almost horizontal. It is known that wire mesh has low strength compared to dense aluminum, such as the precursor at room temperature, but aluminum foam is even lower in strength, and wire mesh can shape aluminum foam [48]. In this study, the wire mesh controlled the vertical foaming of the A1050 and ADC12 precursors, and the joining interface was maintained horizontally.

When the current of the lower halogen lamps II was low (1 A–3 A), the pores of the lower ADC12 foam layer were small, suggesting that the ADC12 foam was in the initial stage of foaming. In contrast, when the current of the lower halogen lamps II was high (4 A–5 A), the pores of the ADC12 foam were fully foamed, and the A1050 foam and ADC12 foam had almost the same pore size, suggesting that they foamed simultaneously. When the current of the lower “Halogen lamps II” was 6 A, the pores were considered to begin coarsening. This may be due to the fact that when heating at a high current value, the temperature rose quickly and reached a high temperature, causing the A1050 foam and ADC12 foam to contact each other due to foaming, resulting in overheating of the entire aluminum foam.

Note that the foaming time *t* became shorter as the current value of the lower halogen lamps II increased. Since the upper “Halogen lamps I” was constant and the heating was stopped after the A1050 foam was observed from the mold, *t* was also expected to be constant. However, since *t* decreased as the current of the lower “Halogen lamps II” increased, it was assumed that the heating from the lower “Halogen lamps II” also had some effects. When the current of the lower “Halogen lamps II” was low, the A1050 foam foamed first, and the ADC12 foam foamed with a delay, so the ADC12 foam was in the initial stage of foaming when the heating was stopped. In contrast, as the current in the lower “Halogen lamps II” increased, the ADC12 foam foamed first, and the A1050 foam foamed later. When the ADC12 foam foamed, it contacted the A1050 precursor from below through the wire mesh, and the precursor was also heated from below by heat transfer, which is considered to have reduced the heating time *t*.

**Figure 10:**
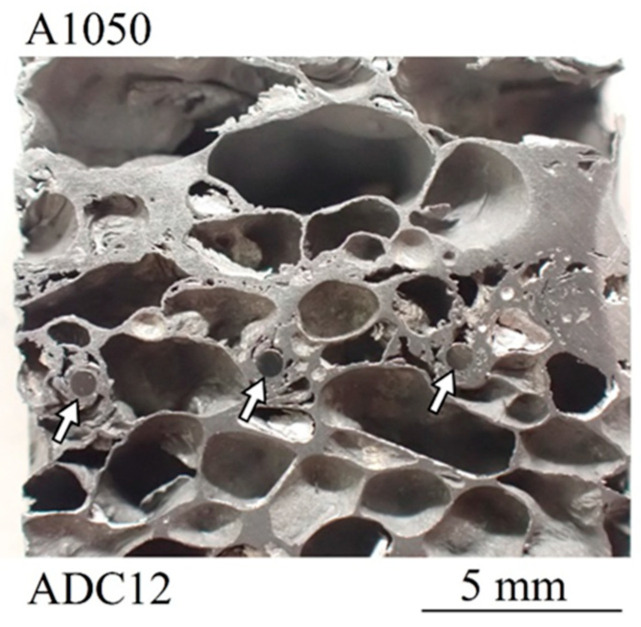
Joining interface of a sample cut near the center of the lower “Halogen lamps II” with a current of 4 A (Figure 9d).

Figure 10 shows an observation of the area near the joining interface of a sample cut near the center of the lower “Halogen lamps II” with a current of 4 A (Figure 9d). No clear joining interface can be seen. The white arrows indicate wire mesh embedded in aluminum foam, and pores can also be observed at the openings of the wire mesh. This is the same tendency as for single samples [61], and it is considered that the compression caused by the pressing process immediately after foaming also filled the openings of the wire mesh with aluminum foam, which broke the oxide film of the two precursors and joined them.

## 6. Conclusions

In this study, we attempted to fabricate a two-layer aluminum foam consisting of an A1050 foam in the upper layer and an ADC12 foam in the lower layer by using an optical heating device that can heat from both the upper and lower sides. The following results were obtained.

(1) By optical heating from both sides and pressing immediately after foaming, both precursors can be foamed and joined, and a two-layer A1050/ADC12 foam can be fabricated.

(2) In the method in which two precursors were stacked and foamed, even the light intensity of the halogen lamps on the top and bottom were adjusted, heat conduction occurred between the stacked precursors and the foaming of each precursor could not be controlled, resulting in tilting of the joining interface.

(3) In the method of foaming using a wire mesh with a gap between two precursors, it was found that by adjusting the light intensity, the two precursors can be foamed almost simultaneously and achieve similar pore structures. The joining interface can also be maintained horizontally.

## Data Availability

Data are contained within the article.
